# Genetic evidence for associations between gastroesophageal reflux disease and site-specific extraesophageal cancers: A Mendelian randomization study

**DOI:** 10.1097/MD.0000000000050874

**Published:** 2026-10-02

**Authors:** Siyu Wang, Menglin Guo, Siyu Zhang, Xiangyu Long, Jing He

**Affiliations:** a Department of Oncology, Sichuan University West China Hospital Guang’an Hospital, Guang’an, Sichuan, China; b Department of Oncology, Sichuan University West China Hospital Jin Cheng Hospital, Chengdu, Sichuan, China; c Department of Oncology, Sichuan Provincial People’s Hospital Jin Niu Hospital, Chengdu, Sichuan, China.

**Keywords:** gastroesophageal reflux disease, lung cancer, Mendelian randomization, pancreatic cancer

## Abstract

Observational studies have suggested an increased risk of extraesophageal cancers in patients with gastroesophageal reflux disease (GERD), but whether these associations are causal remains uncertain. This study systematically evaluated the potential associations between genetically predicted GERD and 22 extraesophageal cancers. Genetic instruments for GERD were obtained from a large genome-wide association study including 129,080 cases and 473,524 controls. Outcome data for 22 site-specific extraesophageal cancers were obtained from multiple genome-wide association study datasets, including the UK Biobank, FinnGen, and international cancer consortia. Two-sample Mendelian randomization (MR) analyses were performed, and dataset-specific MR estimates were subsequently combined using meta-analysis. For positive findings, reverse MR and Multivariable Mendelian randomization analyses were further conducted to assess causal direction and independence from body mass index, smoking intensity, and alcohol consumption. Meta-analysis of two-sample MR showed Bonferroni-significant positive associations of genetically predicted GERD with lung cancer (odds ratio [OR] = 1.220; 95% confidence interval [CI]: 1.100–1.354; *P* = .0002) and pancreatic cancer (OR = 1.517; 95% CI: 1.214–1.895; *P* = .0002). Suggestive positive associations were also observed for laryngeal cancer (OR = 1.774; 95% CI: 1.091–2.884; *P* = .021) and bladder cancer (OR = 1.254; 95% CI: 1.047–1.501; *P* = .014), although neither survived Bonferroni correction. In Multivariable Mendelian randomization analyses accounting for body mass index, smoking intensity, and alcohol consumption, the association with lung cancer remained statistically significant (OR = 1.190; 95% CI: 1.040–1.362; *P* = .012), whereas the associations with pancreatic, laryngeal, and bladder cancers were no longer statistically significant. These findings provide genetic evidence supporting an association between genetically predicted GERD and lung cancer. The evidence for pancreatic, laryngeal, and bladder cancers was less robust and requires further validation in larger independent datasets. These findings may help prioritize future research on cancer risk among individuals with GERD, with further studies warranted to validate the observed associations and clarify their underlying mechanisms.

## Graphical abstract

**Figure:**
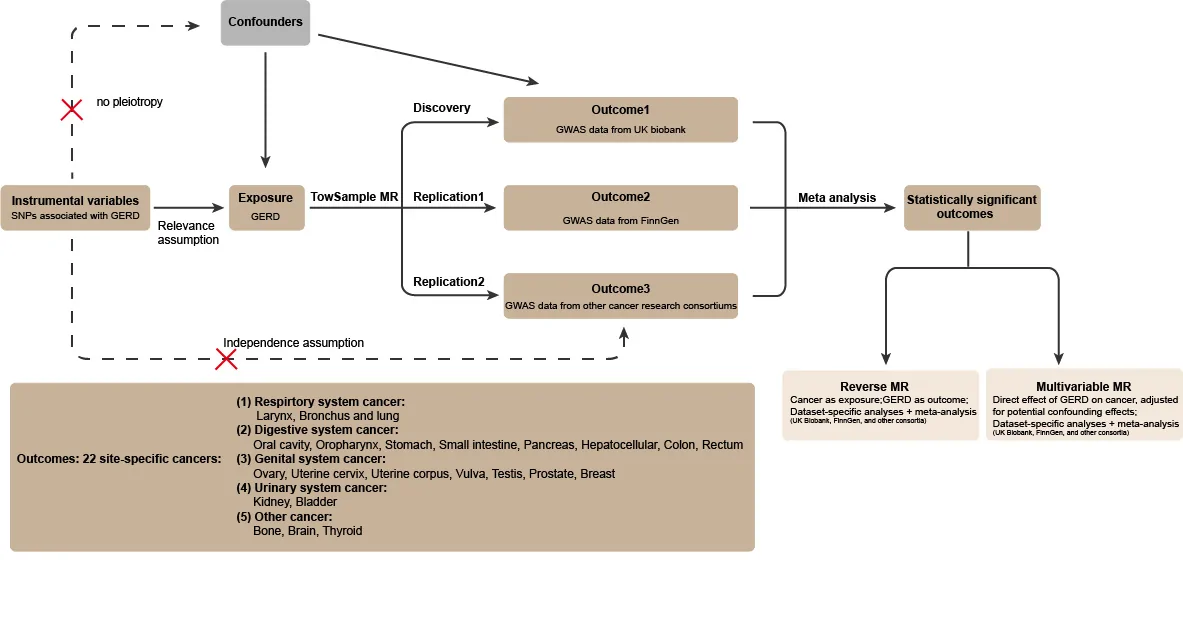

## 1. Introduction

Gastroesophageal reflux disease (GERD) is a common chronic inflammatory condition characterized by reflux of gastric contents into the esophagus, including nonerosive reflux disease, reflux esophagitis, and Barrett’s esophagus.^[1]^ A well-established causal relationship exists between GERD and esophageal adenocarcinoma (EAC) with persistent inflammation, which is considered a key etiological factor. Barrett’s esophagus is widely recognized as a precursor lesion of EAC,^[2–6]^ and individuals with typical reflux symptoms have an approximately fivefold increased risk of developing EAC.^[1]^ Furthermore, various guidelines recommend standardized GERD management and endoscopic surveillance to mitigate the progression of esophageal cancer.^[7–9]^

As a chronic inflammatory disease state, GERD can affect organs beyond the esophagus. Persistent inflammation associated with chronic reflux disease may be accompanied by oxidative stress, immune dysregulation, and metabolic disturbances, which together could contribute to a systemic environment relevant to tumor development.^[10,11]^ Against this biological background, recent observational studies have reported positive associations between GERD and several extraesophageal cancers. For example, a cohort study with a median 9.9-year follow-up found a 5.42-fold higher risk of laryngeal cancer in patients with GERD.^[11]^ A multicenter retrospective case-control study also reported an association with lung cancer risk regardless of treatment,^[12]^ and a Taiwanese cohort study observed an association with colorectal cancer in both sexes.^[13]^ However, these findings remain difficult to interpret causally because observational designs are vulnerable to residual confounding, reverse causation, and potential detection biases. This limitation is particularly important because GERD shares multiple risk factors with cancer (e.g., obesity, smoking, and alcohol use), making complete adjustment challenging. In the absence of expert consensus on screening or surveillance for extraesophageal cancers among patients with GERD and given the growing global burden of GERD,^[14,15]^ indiscriminate cancer screening would impose substantial clinical and economic burdens. Therefore, robust causal evidence is required for risk stratification in this population.

Mendelian randomization (MR) imitates the design of randomized controlled trials and is a novel approach used in epidemiological studies. Using single nucleotide polymorphisms (SNPs) as instrumental variables (IVs) to infer causality, MR minimizes confounding and reverse causation, thereby overcoming the inherent limitations of observational epidemiology in establishing causal relationships between diagnoses.^[16,17]^ Currently, there are several publicly available genome-wide association studies (GWASs) on GERD and various types of tumors, offering ample opportunities for MR analyses. Two prior MR studies indicated that GERD may be associated with an increased risk of lung cancer.^[18,19]^ However, to the best of our knowledge, no comprehensive MR study at the systemic whole-body level has thoroughly examined the causal links between GERD and specific extraesophageal cancers.

This study used MR analyses to systematically evaluate the potential associations between genetically predicted GERD and 22 site-specific extraesophageal cancers. Our findings aim to provide genetic evidence to inform future research on cancer risk among individuals with GERD and to help prioritize associations that warrant further validation and mechanistic investigation.

## 2. Methods

### 2.1. Study design

Figure 1 shows an overview of the study design. Conducted with strict adherence to the STROBE-MR guidelines^[20]^ (Supplementary Table S1, Supplemental Digital Content 1), this research used two-sample MR analysis to evaluate the potential causal link between GERD and 22 distinct site-specific extraesophageal cancers. SNPs associated with GERD were used as IVs to estimate their effects on cancer risk. These cancers are systematically categorized into several classes, including cancers of the respiratory, digestive, genital, and urinary systems, and other sites. To support harmonized two-sample MR and improve statistical power, we prioritized large GWAS resources with clearly defined and comparable phenotype definitions across datasets. Accordingly, the UK Biobank GWAS summary statistics were used for discovery, given their broad cancer coverage, followed by replication in FinnGen and other international cancer research consortia or academic teams. The results across the datasets were then combined using meta-analysis. For statistically significant findings, reverse MR was performed for directionality testing, while Multivariable Mendelian randomization (MVMR) was conducted to evaluate the direct and independent effect of GERD on cancer risk. Results from these additional analyses were also meta-analyzed across the available datasets. This study exclusively utilized publicly available, nonpersonal data; therefore, ethical approval was not required.

**Figure 1.**
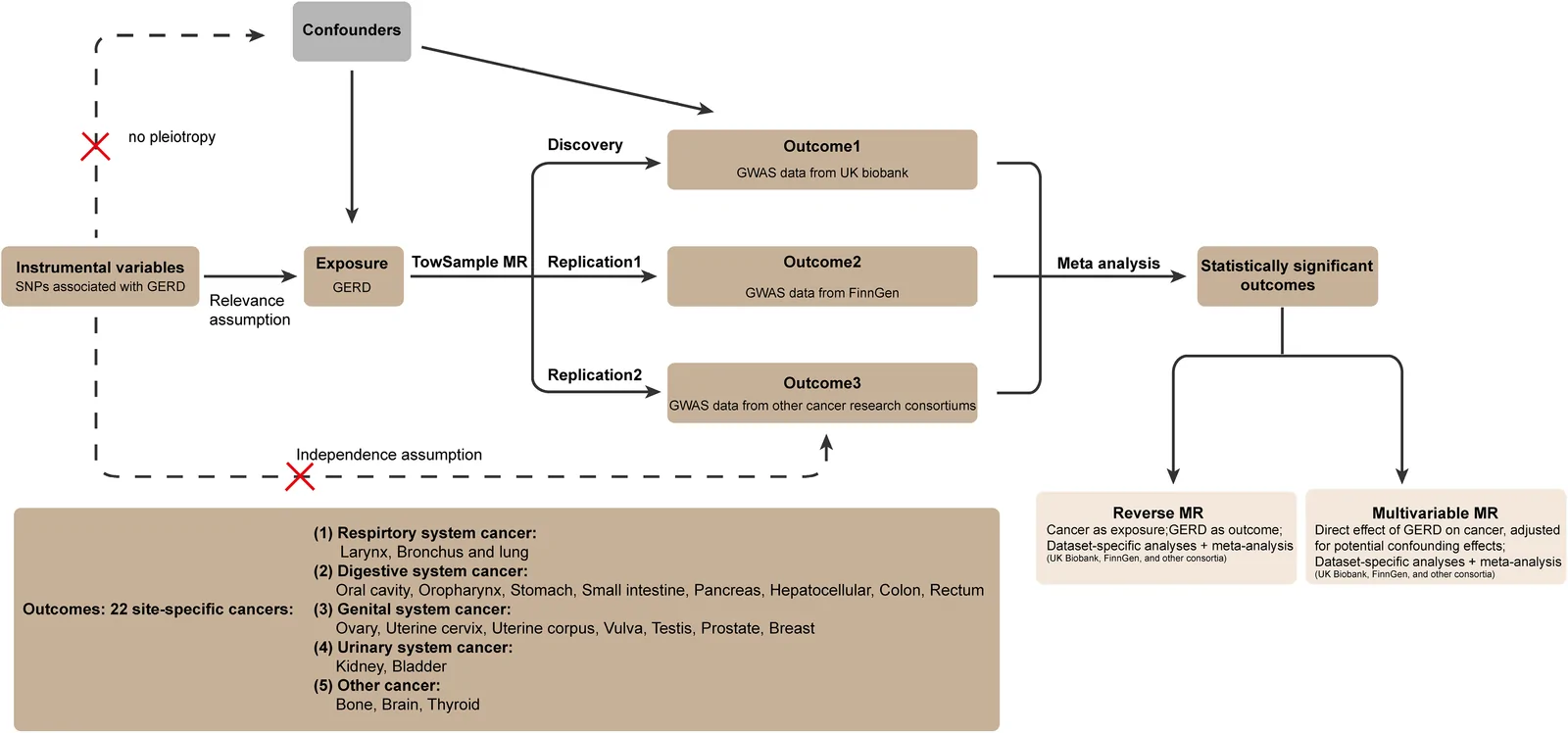
The design of this study. GERD = gastroesophageal reflux disease, MR = Mendelian randomization, SNP = single nucleotide polymorphisms, GWAS = genome-wide association study.

### 2.2. Data sources

The original data regarding GERD were derived from a recent, extensive, and publicly available GWAS conducted by Jue-Sheng Ong et al. This study exclusively included European participants, including 129,080 patients with GERD and 473,524 controls.^[21]^ The corresponding GWAS summary statistics were obtained from the GWAS Catalog (http://ftp.ebi.ac.uk/pub/databases/gwas/summary_statistics/GCST90000001-GCST90001000/GCST90000514). For details on the exposure GWAS, please refer to Supplementary Table S2, Supplemental Digital Content 2.

This study investigated whether GERD is causally associated with 22 types of cancers outside the esophagus. We grouped these cancers into the following categories for easier understanding: tumors of the respiratory system, including laryngeal and lung cancers; tumors of the digestive system, including oral cavity, oropharynx, gastric, small intestinal, pancreatic, hepatic, colonic, and rectal cancers; tumors of the reproductive system, including ovarian, cervical, uterine, vulvar, testicular, prostatic, and breast cancers; tumors of the urinary system, including kidney and bladder cancers; and tumors in other locations, including bone, brain, and thyroid cancers.

GWAS datasets were selected for their maximized statistical power (large sample sizes) and clear, consistent phenotype definitions. We systematically engaged cancer GWAS datasets from 3 pivotal sources, each corresponding to successive stages of our research. Discovery stage (details in Supplementary Table S3, Supplemental Digital Content 3): UK Biobank’s GWAS summary statistical data (https://www.ukbiobank.ac.uk/) were used, given its broad cancer coverage and large sample size. Replication stage 1 (Supplementary Table S4, Supplemental Digital Content 4): For the initial validation, release 9 (r9) from the FinnGen database (https://storage.googleapis.com/finngen-public-data-r9) was used, which provides large-scale GWAS summary statistics under rigorous quality control with consistent phenotype definitions. Replication stage 2 (Supplementary Table S5, Supplemental Digital Content 5): Additional tumor GWAS data from large international cancer research consortia or academic teams were included for further independent replication, leveraging larger case numbers for specific cancer types. This included GWAS data encompassing 15,861 controls and 11,348 cases of lung cancer from the International Lung Cancer Consortium (ILCCO)^[22]^; pancreatic cancer GWAS data from the Pancreatic Cancer Cohort Consortium Genome-Wide Association Study (PanScan1) with 1896 cases and 1939 controls^[23]^; GWAS data involving 40,941 controls and 25,509 cases of ovarian cancer from the Ovarian Cancer Association Consortium^[24]^; a genome-wide meta-analysis of 108,979 controls and 12,906 cases of endometrial cancer by O’Mara et al^[25]^; GWAS data for 61,106 controls and 79,148 cases of prostatic cancer from the Prostate Cancer Association Group to Investigate Cancer-Associated Alterations in the Genome Consortium^[26]^; GWAS data involving 105,974 controls and 122,977 cases of breast cancer from the Breast Cancer Association Consortium^[27]^; and GWAS data involving 431 controls and 649 cases of thyroid cancer, as reported in the study by Kohler A et al^[28]^ Since the UK Biobank database does not include separate GWAS data for oropharyngeal and oral cavity cancers, the GWAS performed by Lesseur et al was used as our discovery cohort.^[29]^

To estimate the direct effect of GERD, body mass index (BMI), smoking intensity, and alcohol consumption were included as additional exposures in the MVMR analyses. Their GWAS summary statistics were obtained from the IEU OpenGWAS project, with details provided in Supplementary Table S6, Supplemental Digital Content 6.

Case numbers varied across outcomes, and a small number of site-specific cancers had relatively few cases in outcome GWAS. For example, laryngeal cancer included only 281 cases in the UK Biobank and 375 cases in FinnGen, whereas small intestinal cancer included only 495 cases in FinnGen. These limited case numbers may have reduced the statistical power and yielded wider confidence intervals (CIs); therefore, the results of these outcomes should be interpreted cautiously.

Potential participant overlap between the exposure and outcome GWAS datasets was assessed based on the reported cohort composition. The GERD GWAS was derived from a large European ancestry meta-analysis that included UK Biobank (UKB). Because only summary-level GWAS data were available, the extent of individual-level overlap could not be directly quantified; therefore, partial overlap with UKB-derived cancer datasets could not be completely excluded. To assess the robustness of the findings and reduce reliance on potentially overlapping UKB estimates, replication analyses were additionally conducted using FinnGen and independent international cancer consortia. For cancer outcomes with multiple data sources, dataset-specific MR estimates were further combined using meta-analysis, and the pooled estimates were used as the primary basis for interpreting the findings.

### 2.3. IV selection

Rigorous MR analysis is based on 3 fundamental assumptions: a strong association between the IVs and the exposure of interest (*P* < 5 × 10^−8^) (Relevance Assumption), the IVs should be unrelated to any confounders related to the outcome (Independence Assumption), and the IVs only influence the outcome through the exposure of interest and not through other pathways (no pleiotropy).^[30,31]^

To identify effective IVs, we initially selected SNPs that were significant across the genome (*P* < 5 × 10^−8^) from the GWAS summary data for GERD. Subsequently, those with linkage disequilibrium were excluded using stringent criteria (genetic distance >10,000 kb and R^2^ < 0.001). Each selected SNP was further examined using PhenoScanner (https://github.com/phenoscanner/phenoscanner), and phenotypic information related to each SNP was recorded (Supplementary Table S7, Supplemental Digital Content 7). SNPs directly related to tumors or known confounding factors for tumor occurrence (such as smoking,^[32,33]^ alcohol consumption,^[34]^ obesity,^[35]^ and diabetes mellitus^[36]^) were excluded to reduce potential horizontal pleiotropy. The excluded SNPs and related phenotypic information are listed in Supplementary Table S8, Supplemental Digital Content 8. This strategy may reduce the number of instruments and thus reduce statistical power. Therefore, robustness was assessed using complementary sensitivity analyses. Finally, to reduce bias introduced by instrument variables, *F* statistics were calculated for each SNP according to the following equation: F = R^2^ × (N–2)/(1–R^2^), where R^2^ represents the proportion of the variance in the exposure variable that is explained by the IVs, and N is the sample size of the exposure in the GWAS study. For the MR analysis, only SNPs for which *F* > 10 were chosen, as genetic variations were considered weak IVs when the *F* statistic was <10.^[37]^ After the corresponding SNPs were extracted from the outcome datasets, effect alleles were harmonized, and ambiguous palindromic SNPs with allele frequencies close to 0.5 were excluded.

### 2.4. Statistical analysis

The R software (version 4.3.2) was used for all analyses, along with the R packages TwoSampleMR (version 0.5.8), MVMR (version 0.4.8), ggplot2 (version 3.5.1), meta (version 7.0.0), and Forestploter (version 1.1.1).

The MR methodology incorporates several approaches: the inverse variance-weighted (IVW) approach, which served as the main evaluative instrument, and the weighted median (WM) and MR-Egger approaches, which were used for sensitivity analysis. IVW assumes that all IVs are valid and generally provides greater statistical power when multiple SNPs are included, but its estimates may be biased in the presence of horizontal pleiotropy. The WM method can provide consistent estimates when more than 50% of the weight is derived from valid IVs. MR-Egger regression, performed under the InSIDE assumption, can yield a valid test of the null hypothesis and a consistent causal estimate even when all variants are invalid IVs, although it is typically less precise and more susceptible to outlying variants.^[38–40]^ The results were quantified using odds ratios (ORs) and 95% CIs to provide a clear statistical representation.

Cochran’s *Q* test was used to assess study heterogeneity. Furthermore, we applied corrections using an IVW random-effects model in cases where the heterogeneity was significant (*P* < .05).^[41,42]^ MR-Egger regression and MR-PRESSO were used to detect horizontal pleiotropy, with the former assessing pleiotropy through the intercept test and the latter identifying outlier SNPs and reestimating the causal effects after outlier removal.^[43,44]^ To ascertain the impact of outlying or pleiotropic SNPs on the inferred causal relationships, each SNP was sequentially excluded from the analysis through a leave-one-out sensitivity analysis, and MR calculations were performed with the remaining SNPs.

Subsequently, a meta-analysis combining data from the discovery and replication stages was performed to integrate and synthesize MR estimates across datasets.^[45,46]^ Fixed-effects models were used when heterogeneity was low (*I*^2^ ≤ 50%), and random-effects models were applied otherwise (*I*^2^ > 50%).^[47]^ The pooled estimates were used as the primary basis for interpreting the findings. When only 1 MR result was available, it was used to interpret the corresponding association. Statistical significance was set at *P* values < .05. To correct for multiple testing across 22 cancer types, Bonferroni correction was applied,^[48]^ with the significance threshold adjusted to .0023 (0.05/22). Associations with *P* values ranging from .0023 to .05 were considered suggestive.

To provide an assessment of statistical power, we performed post hoc power calculations using the online mRnd tool (https://shiny.cnsgenomics.com/mRnd/) for MR power estimation. Power was estimated based on the outcome sample size, case proportion, observed final MR effect estimate, and the variance in GERD explained by the genetic instruments (*R*^2^ = 0.02976), at the nominal significance level (α = .05). Power estimates were classified as adequate (≥0.80), moderate (0.50–0.80), or limited (<0.50).^[49]^

For cancer outcomes showing statistically significant associations with GERD, reverse MR was performed with cancer as the exposure and GERD as the outcome to assess causal direction. Instrument selection and data harmonization followed the procedures described above.^[20]^ To determine whether the observed associations were independent of major shared risk factors, MVMR was conducted by jointly including GERD, BMI, smoking intensity, and alcohol consumption as exposures, thereby estimating the direct effect of GERD on cancer risk after accounting for the other exposures. MVMR-IVW was used as the primary method.^[50]^ Dataset-specific reverse MR and MVMR estimates were subsequently combined using meta-analysis according to the models described above.

## 3. Results

### 3.1. Instrumental variables

Through strict screening, we identified 68 SNPs that served as IVs for GERD. The total variance explained (*R*^2^) by these IVs was 0.02976. Each IV exhibited an *F* statistic exceeding 10, with an average of 191.076 (ranging from 135.880 to 208.004). This indicated the strong validity of the selected IVs. Supplementary Table S9, Supplemental Digital Content 9 provides additional information on the selected IVs.

### 3.2. GERD’s causal effects on cancer risk in the discovery cohort

In our initial investigation of the discovery cohort, we explored the correlation between GERD and 22 site-specific cancers (Table 1, Figure 2, Supplementary Table S10, Supplemental Digital Content 10). Overall, based on the IVW method, GERD showed a Bonferroni-significant positive association with pancreatic cancer (OR = 2.038, 95% CI: 1.367–3.039, *P* = 4.77 × 10^−4^). In addition, GERD showed suggestive positive associations with laryngeal cancer (OR = 2.432, 95% CI: 1.177–5.028, *P* = .016), lung cancer (OR = 1.433, 95% CI: 1.130–1.817, *P* = .003), and bladder cancer (OR = 1.409, 95% CI: 1.105–1.795, *P* = .006); however, these associations did not meet the Bonferroni-corrected threshold.

**Table 1:** Causal links between gastroesophageal reflux disease and 22 site-specific extraesophageal cancers in the UK Biobank (primary results).

Phenotypes	OR_IVW_(95%CI)	*P*_IVW_	*P*_heterogeneity(C_ochran’s *Q*)	*P*_pleiotropy(MR- egger intercept)_	*P*_pleiotropy(MR-PRESSO)_
Respirtory system cancer					
Larynx	2.432 (1.177, 5.028)	.016	.981	.598	.982
Bronchus and lung	1.433 (1.130, 1.817)	.003	.248	.506	.265
Digestive system cancer					
Oral cavity	1.475 (0.960, 2.267)	.076	.443	.201	.453
Oropharynx	1.291 (0.763, 2.185)	.341	.079	.891	.075
Stomach	1.176 (0.757, 1.829)	.471	.514	.432	.519
Small intestine	1.484 (0.608, 3.619)	.386	.056	.267	.052
Pancreas	2.038 (1.367, 3.039)	4.77*10^–4^	.632	.885	.637
Hepatocellular	2.537 (0.855, 7.526)	0.093	0.348	0.771	0.340
Colon	1.112 (0.909, 1.361)	.302	.580	.263	.565
Rectum	1.131 (0.876, 1.460)	.346	.178	.041	.184
Genital system cancer					
Ovary	1.149 (0.761, 1.734)	.510	.057	.038	.064
Uterine cervix	1.328 (0.637, 2.766)	.449	.575	.364	.132
Uterine corpus	0.719 (0.504, 1.025)	.068	.106	.514	.599
Vulva	1.582 (0.542, 4.617)	.402	.854	.515	.853
Testis	1.170 (0.648, 2.114)	.602	.886	.701	.897
Prostate	0.948 (0.806, 1.114)	.515	.136	.597	.163
Breast	1.008 (0.901, 1.127)	.894	.514	.042	.492
Urinary system cancer					
Kidney	1.394 (0.968, 2.006)	.074	.188	.681	.182
Bladder	1.409 (1.105, 1.795)	.006	.979	0.510	.991
Other cancer					
Bone	0.627 (0.244, 1.608)	.331	.401	.981	.397
Brain	1.024 (0.636, 1.649)	.923	.500	.196	.496
Thyroid	1.000 (0.529, 1.890)	.999	.185	.904	.187

CI = confidence interval, IVW = inverse variance-weighted, OR = odds ratio.

**Figure 2.**
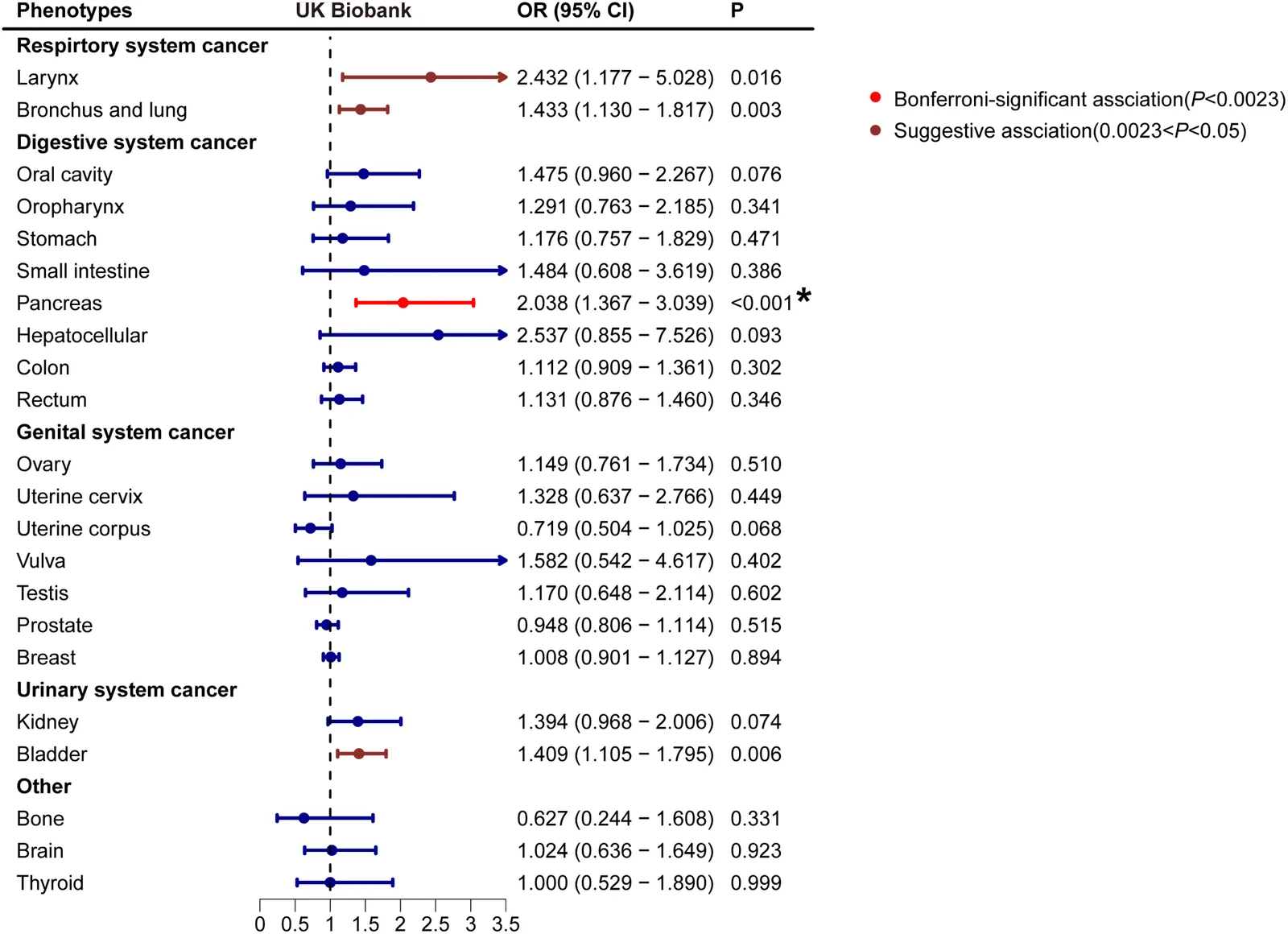
Forest plots showing the association between GERD and extraesophageal cancers in the discovery cohort (primary results). *Bonferroni-significant (*P* < .0023), OR = odds ratio, CI = confidence interval.

For pancreatic and lung cancers, the WM and MR-Egger analyses were directionally consistent with the IVW estimates. For laryngeal and bladder cancers, the MR-Egger estimates were directionally inconsistent with the IVW and WM results. Sensitivity analyses showed no significant evidence of horizontal pleiotropy or heterogeneity based on the MR-Egger intercept, MR-PRESSO, and Cochran’ s *Q* test. Moreover, the SNPs had no significant impact on the observed association between GERD and specific cancers, as indicated by leave-one-out analysis (Supplementary Fig S1, Supplemental Digital Content 11).

No other cancer outcomes met the predefined significance thresholds in the discovery cohort.

### 3.3. GERD’s causal effects on cancer risk in the replication cohort

In the first replication stage, data from 20 cancer GWASs obtained from the FinnGen database were included (Table 2, Supplementary Fig S2, Supplemental Digital Content 12, and Supplementary Table S11, Supplemental Digital Content 13). Overall, the results of the IVW method showed that GERD was suggestively associated with a higher risk of pancreatic cancer (OR = 1.410, 95% CI: 1.027–1.936, *P* = .034). Conversely, GERD was suggestively associated with a lower risk of small intestinal cancer (OR = 0.554, 95% CI: 0.313–0.981, *P* = .034). The results obtained using the WM and MR-Egger method were consistent in direction, with no evidence of pleiotropy or heterogeneity. Additionally, no single IV exerted a significant impact, as shown by the leave-one-out analysis (Supplementary Fig. S1, Supplemental Digital Content 11).

**Table 2:** Causal links between gastroesophageal reflux disease and 22 site-specific extraesophageal cancers in the FinnGen (replication1 results).

Phenotypes	OR_IVW_(95%CI)	*P*_IVW_	*P*_heterogeneity(Cochran’s *Q*)_	*P*_pleiotropy(MR-egger intercept)_	*P*_pleiotropy(MR-PRESSO)_
Respirtory system cancer					
Larynx	1.373 (0.714, 2.641)	.342	.232	.698	.698
Bronchus and lung	1.151 (0.980, 1.352)	.086	.640	.755	.755
Digestive system cancer					
Oral cavity	1.106 (0.701, 1.747)	.664	.200	.749	.749
Oropharynx	/	/	/	/	/
Stomach	1.164 (0.814, 1.662)	.406	.174	.364	.183
Small intestine	0.554 (0.313, 0.981)	.043	.206	.999	.201
Pancreas	1.410 (1.027, 1.936)	.034	.885	1.000	.877
Hepatocellular	0.761 (0.435, 1.333)	.340	.551	.814	.582
Colon	0.841 (0.684, 1.035)	.102	.188	.751	.209
Rectum	1.014 (0.772, 1.332)	.919	.111	.594	.099
Genital system cancer					
Ovary	0.738 (0.491, 1.111)	.145	.131	.441	.134
Uterine cervix	1.425 (0.768, 2.645)	.261	.830	.854	.834
Uterine corpus	0.774 (0.566, 1.058)	.108	.114	.182	.119
Vulva	/	/	/	/	/
Testis	1.091 (0.605, 1.967)	.773	.553	.946	.607
Prostate	1.071 (0.934, 1.227)	.325	.071	.946	.068
Breast	0.971 (0.865, 1.089)	.610	.109	.159	.083
Urinary system cancer					
Kidney	0.830 (0.644, 1.070)	.151	.928	.629	.941
Bladder	1.088 (0.832, 1.422)	.539	.814	.867	.822
Other cancer					
Bone	1.584 (0.693, 3.618)	.275	.839	.312	.860
Brain	0.913 (0.557, 1.498)	.719	.044	.288	.058
Thyroid	0.881 (0.655, 1.185)	.404	.382	.471	.370

CI = confidence interval, IVW = inverse variance-weighted, OR = odds ratio.

In replication stage 2 (Table 3, Supplementary Fig S3, Supplemental Digital Content 14, and Supplementary Table S12, Supplemental Digital Content 15), using GWAS data from the ILCCO database, GERD showed a Bonferroni-significant association with a higher risk of lung cancer (OR = 1.333, 95% CI: 1.110–1.601, *P* = .002). WM and MR-Egger estimates were directionally consistent. However, heterogeneity (Cochran’s *Q*, *P* = .004) and horizontal pleiotropy (MR-PRESSO, *P* = .001) were detected. After the removal of the 4 MR-PRESSO-identified outlier SNPs, the estimate remained in the same direction with a similar magnitude (OR = 1.201, 95% CI: 1.018–1.417, *P* = .030), while heterogeneity (Cochran’s *Q*, *P* = .176) and the MR-PRESSO global test (*P* = .165) were attenuated. Leave-one-out analysis indicated that no single SNP disproportionately influenced the results. Additionally, using GWAS data from O’Mara’s study, GERD was found to be suggestively associated with a higher risk of uterine corpus cancer (OR = 1.151, 95% CI: 1.009–1.312, *P* = .036). The WM and MR-Egger estimates were directionally consistent with the IVW result, with no evidence of heterogeneity or horizontal pleiotropy; leave-one-out analysis indicated that no individual SNP significantly influenced the observed association.

**Table 3:** Causal links between gastroesophageal reflux disease and 22 site-specific extraesophageal cancers in other consortiums (replication2 results).

Phenotypes	OR_IVW_(95%CI)	*P*_IVW_	*P*_heterogeneity(Cochran’s *Q*)_	*P*_pleiotropy(MR-egger intercept)_	*P*_pleiotropy(MR-PRESSO)_
Respirtory system cancer					
Larynx	/	/	/	/	/
Bronchus and lung	1.201 (1.018, 1.417)	.030	.176	.311	.165
Digestive system cancer					
Oral cavity	/	/	/	/	/
Oropharynx	/	/	/	/	/
Stomach	/	/	/	/	/
Small intestine	/	/	/	/	/
Pancreas	1.140 (0.689, 1.886)	.609	.706	.579	.730
Hepatocellular	/	/	/	/	/
Colon	/	/	/	/	/
Rectum	/	/	/	/	/
Genital system cancer					
Ovary	1.035 (0.924, 1.159)	.556	.653	.865	.649
Uterine cervix	/	/	/	/	/
Uterine corpus	1.151 (1.009, 1.312)	.036	.320	.313	.330
Vulva	/	/	/	/	/
Testis	/	/	/	/	/
Prostate	0.969 (0.894, 1.050)	.445	.156	.736	.157
Breast	0.998 (0.931, 1.070)	.953	.065	.125	.074
Urinary system cancer					
Kidney	/	/	/	/	/
Bladder	/	/	/	/	/
Other cancer					
Bone	/	/	/	/	/
Brain	/	/	/	/	/
Thyroid	2.085 (0.830, 5.235)	.118	.162	.053	.145

CI = confidence interval, IVW = inverse variance-weighted, OR = odds ratio.

### 3.4. Meta-analysis and statistical power assessment

In our meta-analysis, we evaluated cancer types for which at least 2 reliable two-sample MR analyses were available, integrating the findings from both discovery and replication cohorts. For cancer types represented by a single credible MR analysis, the results were adopted as the final outcome. The analyses indicated that GERD had a Bonferroni-significant positive causal relationship with 2 specific types of extraesophageal cancer, achieving statistical significance (*P* < .0023). Specifically, lung cancer exhibited a Bonferroni-significant association (OR = 1.220; 95% CI: 1.100–1.354; *P* = .0002), based on 3 combined MR results from the UK Biobank, FinnGen, and ILCCO, and a similar association was noted for pancreatic cancer (OR = 1.517; 95% CI: 1.214–1.895; *P* = .0002), where the analyses incorporated results from 3 MR analyses from the UK Biobank, FinnGen, and PanScan1. Additionally, suggestive positive associations were observed for laryngeal cancer (OR = 1.774, 95% CI: 1.091–2.884; *P* = .021) and bladder cancer (OR = 1.254, 95% CI: 1.048–1.501; *P* = .014), based on MR evidence from both the UK Biobank and FinnGen; however, neither association remained significant after Bonferroni correction. For the remaining cancers, no associations were observed. The pooled MR estimates from the meta-analysis are presented in Table 4 and Figure 3.

**Table 4:** Causal links between gastroesophageal reflux disease and 22 site-specific extraesophageal cancers (meta-analysis).

Phenotypes	Gastroesophageal reflux disease				
	Data sources	OR (95% CI)	*P*	*I* ^2^	Selected model
Respirtory system cancer					
Larynx	**UKB + FinnGen**	**1.774 (1.091, 2.884**)	**.021**	23.9%	Fixed-effect model
Bronchus and lung	**UKB + FinnGen + ILCCO**	**1.220 (1.100, 1.354**)	**.0002**	12.9%	Fixed-effect model
Digestive system cancer					
Oral cavity	Lesseur C + FinnGen	1.289 (0.942, 1.762)	.112	0.0%	Fixed-effect model
Oropharynx	Lesseur C	1.291 (0.763, 2.185)	.341	/	/
Stomach	UKB + FinnGen	1.164 (0.890, 1.523)	.266	0.0%	Fixed-effect model
Small intestine	UKB + FinnGen	0.852 (0.327, 2.219)	.744	69.9%	Random-effects model
Pancreas	**UKB + FinnGen + PanScan1**	**1.517 (1.214, 1.895**)	**.0002**	43.5%	Fixed-effect model
Hepatocellular	UKB + FinnGen	1.265 (0.395, 4.058)	.692	73.1%	Random-effects model
Colon	UKB + FinnGen	0.968 (0.736, 1.273)	.818	72.1%	Random-effects model
Rectum	UKB + FinnGen	1.075 (0.892,1.295)	.449	0%	Fixed-effect model
Genital system cancer					
Ovary	UKB + FinnGen + OCCA	1.019 (0.916, 1.132)	.733	28.2%	Fixed-effect model
Uterine cervix	UKB + FinnGen	1.384 (0.862, 2.221)	.178	0%	Fixed-effect model
Uterine corpus	UKB + FinnGen + O’Mara	0.892 (0.654, 1.218)	.474	79.8%	Random-effects model
Vulva	UKB	1.582 (0.542, 4.617)	.402	/	/
Testis	UKB + FinnGen	1.130 (0.744, 1.715)	.567	0%	Fixed-effect model
Prostate	UKB + FinnGen + PRACTICAL	0.987 (0.926, 1.052)	.688	0%	Fixed-effect model
Breast	UKB + FinnGen + BCAC	0.994 (0.943, 1.048)	.830	0%	Fixed-effect model
Urinary system cancer					
Kidney	UKB + FinnGen	1.057 (0.637, 1.755)	.829	80.9%	Random-effects model
Bladder	**UKB + FinnGen**	**1.254 (1.047, 1.501**)	**.014**	49.1%	Fixed-effect model
Other cancer					
Bone	UKB + FinnGen	1.025 (0.414, 2.540)	.957	52.4%	Random-effects model
Brain	UKB + FinnGen	0.969 (0.687, 1.366)	.857	0%	Fixed-effect model
Thyroid	UKB + FinnGen	0.963 (0.744, 1.246)	.773	34.7%	Fixed-effect model

Bold values indicate statistical significance at *P* < .05.

BCAC = Breast Cancer Association Consortium, C = confidence interval, ILCCO = International Lung Cancer Consortium, OCAC = Ovarian Cancer Association Consortium, OR = odds ratio, PanScan1 = Pancreatic Cancer Cohort Consortium Genome-Wide Association Study, PRACTICAL = Prostate Cancer Association Group to Investigate Cancer-Associated Alterations in the Genome Consortium.

**Figure 3.**
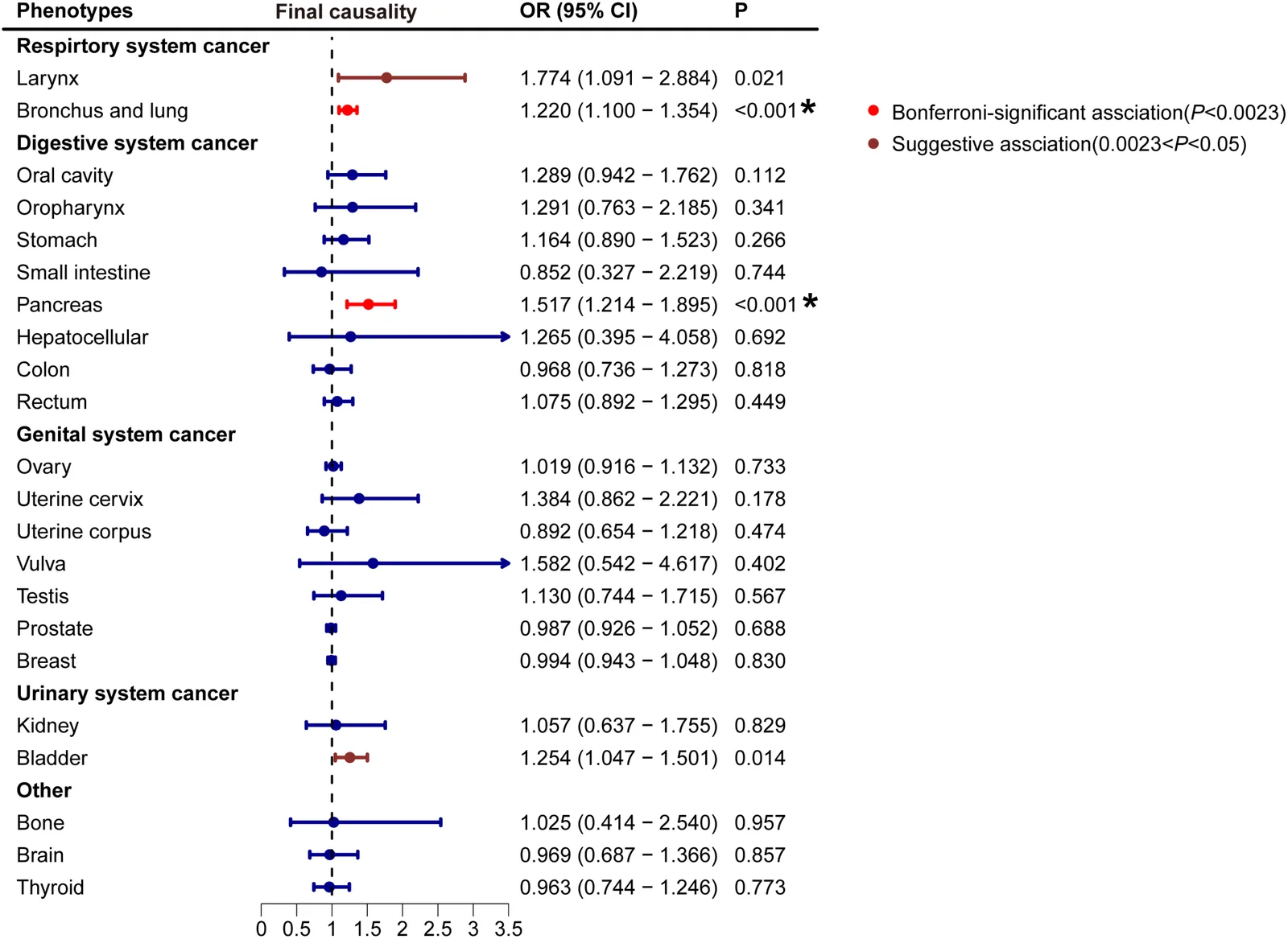
Forest plots showing the final results of the association between GERD and extraesophageal cancers, combining the results from the discovery and replication stages through meta-analysis (final results).*Bonferroni-significant (*P* < .0023), OR = odds ratio, CI = confidence interval.

Statistical power estimates using mRnd are presented in Supplementary Table S13, Supplemental Digital Content 16. Power was high for the Bonferroni-significant associations with lung cancer and pancreatic cancer, both reaching 1.00, and was moderate for the potential associations with laryngeal cancer (0.72) and bladder cancer (0.75). Several null outcomes showed limited post hoc power, such as small intestinal cancer (0.17), hepatic cancer (0.16), and vulvar cancer (0.15).

### 3.5. Reverse and multivariable MR analyses

Reverse MR analyses were conducted for the 4 cancer outcomes that showed either Bonferroni-significant associations (lung and pancreatic cancers) or suggestive associations (laryngeal and bladder cancers) in the primary meta-analysis. Each cancer was treated as the exposure and GERD as the outcome. After pooling the dataset-specific estimates, no evidence was found that genetic liability to any of these cancers causally affected GERD (Supplementary Table S14, Supplemental Digital Content 17).

To determine whether these associations persisted after accounting for BMI, smoking intensity, and alcohol consumption, MVMR analyses were performed for the same 4 cancer outcomes, with GERD and these factors jointly included as exposures (Supplementary Table S15, Supplemental Digital Content 18). Dataset-specific MVMR-IVW estimates were then pooled using meta-analysis. The direct association between GERD and lung cancer remained statistically significant (OR = 1.190, 95% CI: 1.040–1.362; *P* = .012), supporting the robustness of the primary finding. In contrast, no statistically significant direct associations were observed for pancreatic cancer (OR = 1.269, 95% CI: 0.785–2.053; *P* = .331), laryngeal cancer (OR = 1.355, 95% CI: 0.747–2.458; *P* = .317), or bladder cancer (OR = 1.070, 95% CI: 0.844–1.355; *P* = .576) (Table 5).

**Table 5:** Meta-analysis of MVMR estimates of the direct effects of GERD on lung, pancreatic, laryngeal and bladder cancers.

Outcome	Exposure	Method	N	Outcome Datasets	OR (95% CI)	*P*	*I* ^2^	Selected model
Lung cancer	GERD	Meta-analysis of MVMR-IVW direct effects (adjusted for cigarettes smoked per day, drinks per week, and BMI)	3	UK Biobank; FinnGen; ILCCO	**1.190 (1.040, 1.362**)	**.012**	0.0%	Fixed-effect model
Pancreatic cancer			3	UK Biobank; FinnGen; PanScan1	1.269 (0.785, 2.053)	.331	55.1%	Random-effects model
Laryngeal cancer			2	UK Biobank; FinnGen;	1.355 (0.747, 2.458)	.317	0.0%	Fixed-effect model
Bladder cancer			2	UK Biobank; FinnGen;	1.070 (0.844, 1.355)	.576	0.0%	Fixed-effect model

Bold values indicate statistical significance at *P* < .05.

BMI = body mass index, CI = confidence interval, ILCCO = International Lung Cancer Consortium, MVMR = multivariable Mendelian randomization, OR = odds ratio, PanScan1 = Pancreatic Cancer Cohort Consortium Genome-Wide Association Study.

## 4. Discussion

Using a comprehensive two-sample MR framework, we evaluated the potential causal links between GERD and 22 site-specific extraesophageal cancers across the discovery, replication, and meta-analysis stages. The meta-analysis of the two-sample MR demonstrated Bonferroni-significant positive associations between GERD, lung cancer, and pancreatic cancer. The associations with laryngeal and bladder cancers were suggestive but did not survive Bonferroni correction and showed limited robustness in sensitivity analyses. In the MVMR analyses, the direct association between GERD and lung cancer remained statistically significant after accounting for BMI, smoking intensity, and alcohol consumption, further supporting the robustness of this finding. In contrast, no statistically significant direct associations were observed for pancreatic, laryngeal, or bladder cancers. To our knowledge, this is the first study to comprehensively apply MR analysis to determine the causal association between GERD and 22 distinct extraesophageal cancers. Therefore, this study provides initial evidence of GERD’s potential causal relationship in extraesophageal cancer risk.

GERD’s link to EAC is well documented, and clinical guidelines support endoscopic surveillance and screening to enable the early detection of EAC and its precursor lesions, thereby improving outcomes.^[1,3,51,52]^ However, the potential risk of extraesophageal malignancies in patients with GERD may have been underestimated. Several observational studies have identified associations between GERD and various extraesophageal cancers, including malignancies of the head and neck, lung, gastrointestinal tract, and reproductive system. Thus, it is imperative to further investigate and clarify the relationship between GERD and extraesophageal cancer.

For lung cancer, a meta-analysis of two-sample MR estimates from the UK Biobank (3048 cases), FinnGen (5842 cases), and ILCCO (11,348 cases) supported a Bonferroni-significant positive causal association between genetically predicted GERD and lung cancer risk (OR = 1.220, 95% CI: 1.100–1.354; *P* = .0002), consistent with a prior MR study.^[18]^ In the MVMR analysis, this association remained statistically significant after accounting for BMI, smoking intensity, and alcohol consumption (OR = 1.190, 95% CI: 1.040–1.362; *P* = .012), supporting a direct effect of GERD on lung cancer risk independent of these exposures. By integrating 3 large lung cancer GWAS resources and incorporating MVMR, our study extends previous evidence by improving precision and robustness and by evaluating whether the association was independent of major related risk factors. These findings are also in line with previous observational studies that reported an elevated lung cancer risk among individuals with GERD.^[12,53]^

The esophagus is anatomically adjacent to the bronchi and lungs, and pulmonary symptoms such as chronic cough and wheezing are common extraesophageal manifestations of GERD.^[54]^ The potential mechanism by which GERD elevates the risk of lung cancer likely involves chronic microaspiration in GERD patients, leading to sustained pulmonary inflammation. This chronic inflammatory state is associated with increased oxidative stress, which can result in DNA damage, including strand breaks, point mutations, and epigenetic alterations, such as changes in methylation patterns. These changes may subsequently activate oncogenes or inactivate tumor suppressor genes, thereby promoting tumorigenesis.^[55,56]^

Notably, while our MR analysis supports causal inference at the exposure-outcome level, the underlying biological mechanisms remain speculative and require further experimental and clinical investigations.

For pancreatic cancer, a meta-analysis of the two-sample MR estimates from the UK Biobank, FinnGen, and PanScan1 showed a Bonferroni-significant association (OR = 1.517; 95% CI: 1.214–1.895; *P* = .0002). However, after accounting for BMI, smoking intensity, and alcohol consumption, the meta-analyzed MVMR estimate was attenuated and no longer statistically significant (OR = 1.269; 95% CI: 0.785–2.053; *P* = .331), suggesting that the association observed in the two-sample MR analysis may not be fully independent of these risk factors. Thus, the MVMR analysis did not provide evidence for an independent direct effect of GERD on pancreatic cancer risk. Observational evidence linking GERD to pancreatic cancer is relatively limited and sometimes inconsistent. For example, Tran et al reported an inverse association between GERD and pancreatic cancer risk in a longitudinal cohort,^[11]^ whereas Peng et al found a higher pancreatic cancer risk among patients with GERD using proton pump inhibitors, although this study did not directly assess GERD per se.^[57]^ Accordingly, although the two-sample MR meta-analysis supports an association between genetically predicted GERD and pancreatic cancer, whether this association represents an independent direct effect remains uncertain and warrants further investigation in larger independent datasets.

For laryngeal and bladder cancers, the pooled two-sample MR estimates showed suggestive positive associations (laryngeal cancer: OR = 1.774; 95% CI: 1.091–2.884; *P* = .021; bladder cancer: OR = 1.254; 95% CI: 1.047–1.501; *P* = .014), but neither met the Bonferroni-corrected threshold. Moreover, in the discovery stage, the MR-Egger estimates were directionally inconsistent with the IVW and WM estimates, suggesting limited robustness of these associations. Post hoc power was only moderate (0.72 for laryngeal cancer and 0.75 for bladder cancer). The corresponding pooled MVMR estimates were not statistically significant after accounting for BMI, smoking intensity, and alcohol consumption (laryngeal cancer: OR = 1.355; 95% CI: 0.747–2.458; *P* = .317; bladder cancer: OR = 1.070; 95% CI: 0.844–1.355; *P* = .576). Although observational studies have reported an association between GERD and laryngeal cancer,^[58,59]^ residual confounding and other biases remain possible; observational evidence for bladder cancer also exists but is very limited.^[60]^ Taken together, these findings should be considered exploratory and are insufficient to establish causal relationships.

For the remaining cancers, our MR analyses did not provide clear evidence supporting causal associations with GERD. Although previous observational studies have reported possible associations between GERD and cancers such as oral cavity, oropharyngeal, gastric, colon, and prostate cancers, these findings have been inconsistent and may be influenced by residual confounding, reverse causation, or detection bias. In the present MR analysis, most MR effect estimates were close to no effect, with ORs close to 1, and did not reach the prespecified significance threshold. Therefore, these results suggest a lack of clear MR evidence supporting causal associations between GERD and these cancers.

Post hoc power analysis provided additional descriptive information for interpreting these null findings. For cancers with a small number of cases, such as small intestinal, liver, and vulvar cancer, low power estimates may indicate limited statistical power; therefore, the possibility of undetected causal effects cannot be fully excluded. However, low post hoc power should not be attributed simply to insufficient sample size, because the power estimates in this study were calculated based on the observed MR effects. For cancers with large numbers of cases, such as breast, prostate, and colon cancers, low power estimates may mainly reflect that the observed MR effects were close to no effect, with ORs close to 1, rather than being driven solely by an insufficient number of cases. Therefore, post hoc power analysis should be regarded as supplementary descriptive information for interpreting the results, rather than as a primary criterion for determining the presence or absence of causal associations.

MR analyses have yielded inconsistencies in the causal relationship between GERD and certain cancers. For instance, GERD had no clear association with small intestinal cancer in the UK Biobank, whereas a potential association was identified in FinnGen data (OR = 0.554, 95% CI: 0.313–0.981, *P* = .034). Several factors could explain this discrepancy. Although this study used GWAS databases that mainly included populations of European ancestry, the genetic composition varied across the cohorts. The UK Biobank primarily consists of participants from the UK with a relatively homogeneous genetic background, but also includes individuals from other ethnicities, increasing genetic diversity. In contrast, the FinnGen dataset is derived from a Finnish population that exhibits a highly homogeneous genetic background owing to a genetic bottleneck effect and relative isolation. This homogeneity may yield clearer genetic signals. However, some variants may be rare or absent in other European populations, limiting the generalizability of the FinnGen findings. Moreover, differences in cancer phenotype definitions, case composition, sample size, and case numbers across GWAS datasets may also affect the precision and comparability of MR estimates. To reduce reliance on any single data source, results from multiple independent GWAS datasets were compared and, where available, combined using meta-analysis. Nevertheless, these between-dataset differences should be considered when interpreting the pooled findings.

To minimize confounding and other biases, we applied multiple control strategies. SNPs were selected from the GERD GWAS at genome-wide significance (*P* < 5 × 10^−8^), pruned for linkage disequilibrium (genetic distance > 10,000 kb, *R*^2^ < 0.001), and screened using PhenoScanner to exclude variants directly associated with cancer (e.g., rs12357321 linked to ovarian cancer). Given the complexity of cancer etiology, we also excluded SNPs related to established carcinogenic factors, including smoking and alcohol consumption (e.g., rs129678553 and rs2240326), as well as other major risk factors such as obesity. Although this stringent strategy may reduce the number of instruments and statistical power, robustness was further evaluated using complementary sensitivity analyses, including the WM method, MR-Egger method, and MR-PRESSO outlier removal when pleiotropy was detected. To reduce reliance on a single data source, the analyses were replicated across multiple independent GWAS datasets, and dataset-specific MR estimates were combined using meta-analysis. For positive associations identified in the primary analyses, reverse MR was performed to assess causal direction and potential reverse causation, while MVMR was conducted to evaluate whether these associations were independent of BMI, smoking intensity, and alcohol consumption; estimates from these analyses were also meta-analyzed across available datasets. Overall, our study was strengthened by the comprehensive MR assessment of GERD across 22 extraesophageal cancers, rigorous instrument selection and sensitivity analyses, validation across 3 independent GWAS sources with meta-analysis, and additional reverse MR and MVMR analyses for positive findings. Together, these approaches reduced potential bias and improved the precision, reliability, and assessment of the directionality and independence of the observed associations.

However, this study has some limitations. First, the study cohort was largely of European descent, which restricts the applicability of the findings to other demographic groups. Future studies should include more diverse populations in order to verify these results. Second, although we assessed GERD’s overall relationship with 22 types of cancer, we did not conduct detailed analyses of the pathological subtypes of each cancer, thereby limiting the granularity of our findings. Third, although pleiotropy-robust methods and outlier diagnostics were applied, and additional reverse MR and MVMR analyses were performed to assess causal direction and independence, residual horizontal pleiotropy through unmeasured or unknown pathways cannot be completely excluded. Fourth, the GERD exposure GWAS was derived from a meta-analysis of multiple cohorts, including UK Biobank participants. Therefore, potential participant overlap between the GERD exposure GWAS and UK Biobank-derived cancer outcome GWAS cannot be completely excluded. As only summary-level data were available, the extent of individual-level overlap could not be directly quantified. In addition, the outcome GWAS datasets were derived from the UK Biobank, FinnGen, and several international cancer consortia and differed in population characteristics, cancer phenotype definitions, case composition, sample size, and case numbers, which may have affected the precision and comparability of dataset-specific and pooled MR estimates. Although independent replication datasets and meta-analysis were used to reduce reliance on any single data source, these sources of heterogeneity remain a potential limitation. Fifth, limited statistical power is present for some cancers with small case numbers in the outcome GWAS, resulting in wider CIs and potentially unstable estimates.

## 5. Conclusion

This comprehensive MR study systematically evaluated the associations between genetically predicted GERD and 22 site-specific extraesophageal cancers. The primary MR meta-analysis identified Bonferroni-significant positive associations with lung and pancreatic cancers, as well as suggestive positive associations with laryngeal and bladder cancers. However, after accounting for BMI, smoking intensity, and alcohol consumption in MVMR analyses, only the association with lung cancer remained statistically significant. Overall, our findings provide genetic evidence supporting an association between GERD and lung cancer, whereas the evidence for pancreatic, laryngeal, and bladder cancers remains less robust. Further studies in larger, independent, and more diverse populations are warranted to validate these associations and clarify their underlying mechanisms.

## Author contributions

**Data curation:** Siyu Wang, Menglin Guo, Siyu Zhang.

**Formal analysis:** Siyu Wang.

**Investigation:** Siyu Wang.

**Methodology:** Siyu Wang.

**Software:** Siyu Wang.

**Validation:** Siyu Wang.

**Visualization:** Siyu Wang.

**Writing – original draft:** Siyu Wang.

**Conceptualization:** Jing He.

**Writing – review & editing:** Xiangyu Long, Jing He.




































